# Dissociative symptoms mediate the relation between PTSD symptoms and functional impairment in a sample of military members, veterans, and first responders with PTSD

**DOI:** 10.1080/20008198.2018.1463794

**Published:** 2018-05-17

**Authors:** Jenna E. Boyd, Alina Protopopescu, Charlene O’Connor, Richard W. J. Neufeld, Rakesh Jetly, Heather K. Hood, Ruth A. Lanius, Margaret C. McKinnon

**Affiliations:** a Department of Psychology, Neuroscience and Behaviour, McMaster University, Hamilton, Canada; b Mood Disorders Program, St. Joseph’s Healthcare Hamilton, Hamilton, Canada; c Homewood Research Institute, Guelph, Canada; d Program for Traumatic Stress Recovery, Homewood Health Centre, Guelph, Canada; e Department of Neuroscience, Western University, London, Canada; f Department of Psychiatry, Western University, London, Canada; g Department of Psychology, Western University, London, Canada; h Defence Research and Development Canada, Toronto, Canada; i Department of Psychiatry, University of Ottawa, Ottawa, Canada; j Department of Psychiatry and Behavioural Neurosciences, McMaster University, Hamilton, Canada; k Imaging Division, Lawson Health Research Institute, London, Canada

**Keywords:** Functional impairment, dissociative subtype, derealization, PTSD, military members, veterans, first responders, • Posttraumatic Stress Disorder (PTSD) is associated with reduced day-to-day functioning across multiple important life domains, including interpersonal relationships and occupational or educational roles.• This pattern is apparent among military members, veterans, and first responders with PTSD.• This study aimed to gain a better understanding of the symptoms associated with this reduced functioning.• The results of the study indicate that symptoms of dissociation, particularly derealization (feeling as though things around you are unreal or unfamiliar), accounts for the relation between PTSD symptoms and impairment in day-to-day functioning• This work is important in improving both symptom and functional recovery from PTSD among military members and first responders.

## Abstract

**Background**: Posttraumatic Stress Disorder (PTSD) is associated with significant functional impairment in important areas, including interpersonal relationships and occupational or educational roles. Preliminary evidence suggests that the dissociative subtype of PTSD (PTSD+DS), characterized by marked symptoms of depersonalization and derealization, is associated with increased functional impairment and disease severity, including among military members and veterans diagnosed with PTSD. Similarly, first responders (e.g. police, fire, paramedics) have also been found to experience dissociative symptoms. Despite these findings, little work has investigated whether dissociative symptoms are related to heightened functional impairment among these populations.

**Objective**: We examined the relation between functional impairment and symptom level variables, including dissociative symptoms of depersonalization and derealization among military members, veterans, and first responders with probable PTSD. We further investigated the hypothesis that dissociative symptoms mediate the relation between PTSD symptomatology and functional impairment.

**Method**: Eighty-one medical charts of inpatients at a residential PTSD treatment programme were accessed via retrospective review. Sixty-two were included in the present analyses. Comparison of means on symptom measures between first responders and military members/veterans were conducted, followed by correlational and mediation analyses.

**Results**: Compared with first responders, military members and veterans showed higher levels of derealization, functional impairment, alexithymia, anxiety, and depression. Within the total sample, dissociative symptoms emerged as the strongest correlate of functional impairment and, among the dissociative symptom clusters, derealization symptoms demonstrated the strongest relation with impairment. Mediation analyses revealed that total dissociative symptoms and derealization symptoms significantly mediated the relation between PTSD symptoms and functional impairment.

**Conclusions**: These findings highlight the importance of assessing and treating dissociative symptoms, consistent with the dissociative subtype of PTSD, among military members, veterans, and first responders with PTSD. Successful recovery on a functional and symptomatic level may necessitate treatment of dissociative symptoms, particularly derealization.

## Los Síntomas disociativos median la relación entre los síntomas de TEPT y el deterioro funcional en una muestra de miembros militares, veteranos y profesionales en intervención inmediata con TEPT

**Planteamiento**: El trastorno por estrés postraumático (TEPT) se asocia con un deterioro funcional significativo en áreas importantes, incluidas las relaciones interpersonales y los roles ocupacionales o educativos. La evidencia preliminar sugiere que el subtipo disociativo de TEPT (TEPT + SD), caracterizado por síntomas marcados de despersonalización y desrealización, se asocia con mayor deterioro funcional y gravedad de la enfermedad, incluso entre miembros militares y veteranos diagnosticados con TEPT. Del mismo modo, también se ha encontrado que los profesionales en intervención inmediata (por ejemplo, policías, bomberos, paramédicos) también experimentan síntomas disociativos. A pesar de estos hallazgos, pocos han investigado si los síntomas disociativos están relacionados con una mayor deficiencia funcional entre estas poblaciones.

**Objetivo**: Examinamos la relación entre el deterioro funcional y las variables de nivel de síntomas, incluyendo síntomas disociativos de despersonalización, desrealización entre miembros militares, veteranos y profesionales en intervención inmediata con un probable TEPT. Además investigamos la hipótesis de que los síntomas disociativos median la relación entre la sintomatología del TEPT y el deterioro funcional.

**Método**: Se accedió a ochenta y un historiales clínicos de pacientes hospitalizados en un programa residencial de tratamiento de TEPT mediante un análisis retrospectivo. Sesenta y dos fueron incluidos en el presente análisis. Se realizó una comparación de los medios de medición de síntomas entre los profesionales en intervención inmediata y los militares y veteranos, seguida de análisis de correlación y mediación.

**Resultados:** En comparación con los profesionales en intervención inmediata los miembros del ejército y los veteranos mostraron niveles más altos de desrealización, deterioro funcional, alexitimia, ansiedad y depresión.Dentro de la muestra total, los síntomas disociativos surgieron como el correlato más fuerte del deterioro funcional y, entre los grupos de síntomas disociativos, los síntomas de desrealización mostraron la relación más fuerte con el deterioro. Los análisis de mediación revelaron que los síntomas disociativos totales y los síntomas de desrealización mediaban significativamente en la relación entre los síntomas de TEPT y el deterioro funcional.

**Conclusiones**: Estos hallazgos resaltan la importancia de evaluar y tratar los síntomas disociativos, coincidentes con el subtipo disociativo del TEPT, entre los militares, los veteranos y los profesionales en intervención inmediata con TEPT. El éxito de su recuperación a nivel funcional y sintomático puede requerir el tratamiento de los síntomas disociativos, en particular, la desrealización.

## 在军人，退伍老兵和现场急救员样本中分离症状中介了PTSD症状和功能障碍之间的关系

**背景**：创伤后应激障碍（PTSD）与重要方面的明显功能障碍相关：包括人际关系和职业或教育。PTSD（PTSD + DS）的分离亚型以人格解体和现实解体症状为特征。初步证据表明，被诊断为PTSD的军人和退伍老兵中，功能受损和疾病严重程度增加与分离症状有关。同样，现场急救人员（例如警察，火警，医务人员）也被发现患有分离症状。尽管有这些发现，但很少研究调查分离症状是否与这些人群中功能受损加剧相关。

**目标**：我们研究了可能发生的军人、退伍老兵和现场急救人员的功能障碍和症状水平之间

**的关系**：包括人格解体，现实解体，情绪抑制，走神，记忆解离和身份分离等症状。我们进一步考察了分离症状中介PTSD症状和功能障碍之间关系的假设方法：回溯性回顾了一个住院PTSD治疗计划的81位住院病人的医疗图表，62名包括在了目前的分析中。现场急救人员与军人/退伍军人症状水平进行平均值比较，然后进行相关分析和中介分析。

**结果**：与现场救援人员相比，军人和退伍老兵表现出较高水平的现实解体、功能障碍、述情障碍、焦虑和抑郁。在总样本中，分离症状是功能障碍最强的相关因子；并且在分离症状群中，人格解体症状表现出与损伤关系最强的关系。 调节分析显示总分离症状和人格解体症状显着中介了PTSD症状和功能障碍之间的关系。

**结论**：这些发现强调了在军人，退伍老兵和患有PTSD的现场急救人员评估和治疗分离症状，与PTSD的分离亚型一样重要。 在功能和症状水平上成功恢复可能需要治疗分离症状，特别是现实解体症状。

## 1. Introduction

Posttraumatic Stress Disorder (PTSD) is associated with significant distress or impairment in day-to-day functioning in important areas such as family relationships, occupational roles, and educational settings (American Psychiatric Association, 2013). Indeed, PTSD is related to large reductions in work and mental health related quality of life as compared to anxiety disorders (Olatunji, Cisler, & Tolin, 2007), significantly impaired workplace performance and productivity (Kessler, 2000), and the highest use of medical care services among DSM-IV anxiety disorders (Greenberg et al., 1999). In addition, recent work suggests that reduced quality of life may persist following remission of PTSD symptoms. For example, Westphal et al. (2011) reported that individuals with a previous diagnosis of PTSD who did not currently meet criteria for the disorder experienced reduced quality of life when compared to trauma-exposed controls. Despite these findings, limited research has sought to identify symptom level factors predictive of functional impairment among individuals with PTSD – knowledge that is central to tailoring treatment to promote both symptomatic and functional recovery (Lanius, 2015).

The present study sought to identify trauma-related symptoms most strongly associated with functional impairment in a sample of military members, veterans, and first responders (e.g. police, fire, paramedics) with probable PTSD.

### 1.1. Functional impairment among military members, veterans, and first responders with PTSD

The study of functional impairment may be particularly relevant among veteran samples, where individuals with PTSD experience poorer health functioning, increased disability (Goldberg et al., 2014), greater functional impairment, and reduced quality of life compared to veterans without PTSD (Shea, Vujanovic, Mansfield, Sevin, & Liu, 2010; Zatzick et al., 1997). Moreover, among trauma-exposed men, combat trauma has been related to greater incidence of PTSD, unresolved PTSD symptoms, and unemployment (Prigerson, Maciejewski, & Rosenheck, 2001). For example, men who identified combat trauma as their worst lifetime event were more likely to meet lifetime criteria for PTSD, have ongoing PTSD symptoms, and unemployment when compared to men who identified other traumatic experiences as their most disturbing life event (e.g. physical assault), with the exception of sexual assault or molestation (Prigerson et al., 2001). Similarly, first responders involved in rescue and recovery following disasters or terrorist attacks also experience significant functional impairment (North et al., 2002; Ruggero et al., 2013). To illustrate, firefighters who were diagnosed with PTSD following a shared traumatic experience (the Oklahoma City Bombing) reported greater interference in their daily activities, relationships, and decreased job satisfaction in comparison to firefighters who were exposed to the same traumatic event but did not have PTSD (North et al., 2002).

In addition, reported levels of exposure to childhood abuse are significantly higher among military members and veterans when compared with the general population (Afifi et al., 2016; McCauley, Blosnich, & Dichter, 2015). Importantly, childhood abuse is associated with suicide and suicide attempts (Afifi et al., 2016), high utilization of mental health care services and perceived need for mental health care (Turner et al., 2017), and reduced health-related quality of life (Katon et al., 2015) among military members. Although less research has been conducted among first responders, emerging work suggests that early physical abuse is related to worse mental health outcomes in these populations (Komarovskaya et al., 2014). These findings further highlight the importance of understanding functional impairment among military and first responder populations.

### 1.2. The link between functional impairment and PTSD symptomatology

Research investigating which PTSD symptom clusters are associated with the highest level of functional impairment has yielded mixed findings. Here, a small number of studies have found that among PTSD symptoms, emotional numbing/avoidance symptoms as per DSM-IV-TR PTSD diagnostic criteria (American Psychiatric Association, 2000) are most predictive of functional impairment among veterans and active duty military members (Rona et al., 2009; Shea et al., 2010) and among individuals exposed to terrorism (i.e. World Trade Center attacks; Malta, Levitt, Martin, Davis, & Cloitre, 2009). By contrast, other work has identified hyperarousal symptoms as the most significant correlate of functional impairment among military personnel (Maguen, Stalnaker, McCaslin, & Litz, 2009) and natural disaster survivors (Heir, Piatigorsky, & Weisæth, 2010). In other studies, symptoms of emotion dysregulation (e.g. ability to regulate negative mood) and interpersonal difficulties, consistent with the *Diagnostic and Statistical Manual of Mental Disorders, Fifth Edition* (DSM-5; American Psychiatric Association, 2013) conceptualization of PTSD, appear predictive of functional disability among victims of terrorism (Malta et al., 2009) and women with a history of childhood abuse (Cloitre, Miranda, Stovall-McClough, & Han, 2005). For example, Cloitre et al. (2005) reported that measures of interpersonal functioning and emotion regulation were significant predictors of functional impairment in women with a history of childhood abuse and made a contribution to functional impairment equal to that of PTSD symptoms.

### 1.3. PTSD, functional impairment, and dissociation: a probable link

Importantly, much of the work to date has investigated symptoms of PTSD as they were conceptualized in the DSM-IV-TR. DSM-5 brought about a new conceptualization of PTSD symptom clusters, including re-experiencing (e.g. intrusive thoughts, flashbacks), avoidance of trauma-related stimuli, negative alterations in mood and cognition (including emotion regulation difficulties), and alterations in arousal or reactivity (e.g. hypervigilance, increased startle response; American Psychiatric Association, 2013). DSM-5 also introduced the dissociative subtype of PTSD (PTSD+DS), reflecting approximately 15–30% of individuals with PTSD who experience significant dissociative symptoms of depersonalization (feeling as though one is separated from one’s own body) and derealization (feeling as though things around you are strange or unfamiliar) (Armour, Karstoft, & Richardson, 2014; Bennett, Modrowski, Kerig, & Chaplo, 2015; Blevins, Weathers, & Witte, 2014; Bremner & Brett, 1997; Bremner et al., 1992; Frewen, Brown, Steuwe, & Lanius, 2015; Hansen, Ross, & Armour, 2017; Lanius, Brand, Vermetten, Frewen, & Spiegel, 2012; Lanius et al., 2010; Spiegel et al., 2013; Steuwe, Lanius, & Frewen, 2012; Tsai, Armour, Southwick, & Pietrzak, 2015; Waelde, Silvern, & Fairbank, 2005; Wolf et al., 2012, 2012). Among military members and veterans, recent studies indicate that 8–32% of veterans and active duty military personnel meet criteria for the dissociative subtype (Armour et al., 2014; Tsai et al., 2015; Waelde et al., 2005; Wolf et al., 2012, 2012). Critically, within military and veteran samples, as well as other trauma-exposed samples (e.g. incarcerated youth, individuals exposed to motor vehicle accidents, physical or sexual assault, childhood abuse), PTSD+DS has been associated with greater PTSD symptom severity, depressive and alcohol abuse symptoms, and psychiatric comorbidity (Bennett et al., 2015; Blevins et al., 2014; Tsai et al., 2015; Waelde et al., 2005; Wolf et al., 2012, 2012) (but see Steuwe et al. (2012)). Although considerably less work has been done in first responder populations, early work has identified significantly greater levels of dissociative symptoms among police officers with PTSD as compared to those without PTSD (Carlier, Lamberts, Fouwels, & Gersons, 1996). In addition, recent work has identified the presence of peritraumatic dissociation in first responder samples, which has been associated with a greater likelihood of developing PTSD (Galatzer-Levy, Madan, Neylan, Henn-Haase, & Marmar, 2011; Maia et al., 2011; Marmar et al., 2006; van der Velden et al., 2006).

Dissociative symptoms have been related to functional indicators among additional trauma-exposed samples. For example, among a subgroup of men seeking treatment for alcohol dependence who had a lifetime diagnosis of PTSD, those with dissociative symptoms experienced greater interference in quality of life (Evren et al., 2011). Notably, in the total study sample, dissociation mediated the relation between childhood abuse and PTSD severity, and individuals with dissociative symptoms had higher levels of childhood abuse. Further, childhood trauma was also related to worse quality of life, suggesting that experiences of childhood trauma and dissociative symptoms may interact to lead to reduced quality of life or functional impairment (Evren et al., 2011).

### 1.4. Dissociation is associated with poor cognitive functioning in PTSD

Dissociative symptoms are associated with worse cognitive functioning across domains of memory, attention, and executive functioning among individuals with PTSD (see McKinnon et al., 2016, for a review), including veterans (Morgan, Doran, Steffian, Hazlett, & Southwick, 2006; Roca, Hart, Kimbrell, & Freeman, 2006). Notably, cognitive impairment has been associated with reduced psychosocial functioning (Jaeger et al., 2006) and decreased ability to carry out instrumental activities of daily living (McCall & Dunn, 2003) among individuals with depression, even after accounting for depressive symptoms (Jaeger et al., 2006). Similarly, within PTSD samples, reduced verbal memory performance and executive functioning difficulties have been associated with reduced psychosocial functioning (Ainamani, Elbert, Olema, & Hecker, 2017; Geuze et al., 2009; but see Twamley et al., 2009). Although no work to date has illustrated a three-way relation between dissociative symptoms, cognitive dysfunction, and functional impairment in individuals with PTSD, it is probable that the heightened level of cognitive dysfunction seen among individuals with dissociative symptoms and PTSD may be related to worse functional impairment compared to those without dissociative symptoms.

### 1.5. Objectives of the present study

Given that, among individuals with PTSD, dissociative symptoms are associated with heightened disease severity (Stein et al., 2013) and cognitive dysfunction (McKinnon et al., 2016) that together contribute to functional impairment in related disorders (e.g. depression; Gonda et al., 2015), we hypothesized that when compared to classical symptoms of PTSD (e.g. re-experiencing, hyperarousal), dissociative symptoms will contribute most strongly to functional impairment in this population. Accordingly, the present study sought to examine whether dissociative symptoms were significantly associated with functional impairment among military personnel, veterans, and first-responders with PTSD. We also investigated the relation between functional disability and classic PTSD symptom clusters, as well as with additional symptoms associated with PTSD. In order to test our main hypothesis, mediation analyses were used to determine whether dissociative symptoms mediate the relation between PTSD symptoms and functional impairment in this population. In order to determine if the first responder and military/veteran samples in our study experienced similar severity of symptomatology across clinical and functional measures and similar levels of exposure to adverse experiences in childhood, we compared symptom means and reported exposure to adverse childhood experiences (ACEs) between these two groups. Finally, due to the potential impact of childhood abuse exposure on functional outcomes, we conducted subsequent analyses including exposure to ACEs as a covariate in these models.

## 2. Methods

### 2.1. Participants

This study was approved by the Homewood Health Centre Research Ethics Board.

Eighty-one charts were accessed via retrospective chart review of patients seen at Homewood Health Centre’s inpatient Program for Traumatic Stress Recovery between 22 May 2015 and 30 June 2016. Participants were included if they had a score above the proposed cut-point for probable diagnosis of PTSD (score of 33; Wortmann et al., 2016) on the PTSD Checklist for DSM 5 (PCL-5) (*n *= 4 excluded) (Weathers et al., 2013). Participants excluded from the analyses based on missing or incomplete data (*n *= 12; missing data from any variable in the main analyses) or multiple admissions within the study period (*n *= 3), leaving a final sample of 62 patients. The patient sample was comprised of military members and veterans (*n* = 32), first responders (e.g. police, fire fighters, paramedics) (*n = *27) and individuals who were/had previously been employed as both first responders, and military members or veterans (*n* = 3) who were suffering from trauma-related psychological difficulties. Demographic characteristics are reported in Table 1. Secondary analyses investigating exposure to ACEs were completed on a subset of the full sample (*n* = 59), as three respondents had missing data on a measure of ACEs.

**Table 1.** Demographic characteristics of the study sample.

Characteristics	Combined sample, *n* = 62	Military and veteran, *n* = 32	First Responder, *n = *27
*Demographic Characteristics*	Mean (*SD*)		
Sex (female:male)	10:52	7:25	2:25
Age	45.9 (9.5)	45.2 (11.1)	47.3 (7.3)
Education	% of Sample		
8^th^ grade or less	1.6	3.1	0
Some high school	4.8	6.3	0
High school	16.1	21.9	11.1
Technical or trade school	12.9	18.9	7.4
Some college or university	25.8	28.1	25.9
Diploma or bachelor’s degree	32.3	12.5	51.9
Graduate degree	6.5	9.4	3.7
Income	% of Sample		
Employed	59.7	40.6	77.8
Employment Insurance	8.1	3.1	14.8
Pension	19.4	31.3	3.7
Disability Insurance	25.8	28.1	25.9
Other (e.g. Investment, WSIB, Inheritance)	8.1	9.4	7.4
No Income	3.2	6.3	0

### 2.2. Materials

*The World Health Organization Disability Assessment Schedule 2.0* (WHODAS; Üstün, 2010)

The WHODAS is a 36-item self-report inventory used to assess functional disability across six domains with high internal consistency; cognition (chronbach’s alpha (α) = 0.94; understanding and communicating), mobility (α = 0.96), self-care (α = 0.95) (e.g. personal hygiene), getting along (α = 0.94) (interacting with others), life activities (α = 0.94) (e.g. work or home responsibilities), and participation in society (α = 0.95) (engaging in community, civil, and recreational activities). The WHODAS showed good test-retest reliability, and the WHODAS domain scores showed good convergence with comparable instruments that measure disability (Üstün, 2010).

*The PTSD Checklist for DSM-5* (PCL-5; Weathers et al., 2013)

The PCL-5 is a 20-item self-report questionnaire used to assess the severity of PTSD symptoms across the four symptom domains outlined in the DSM-5 with good- high internal consistency (Bovin et al., 2016). It contains items that assess intrusive symptoms (PCL Intrusions; α = 0.80–0.92), avoidance (PCL avoidance; α = 0.83–0.92), negative alterations in mood and cognition (PCL mood and cognition; α = 0.82–0.89), and alterations in arousal and reactivity (PCL arousal and reactivity; α = 0.75–0.84) (α for the total score = 0.91–0.95). The PCL-5 has also been found to have good test-retest reliability, convergent validity, and sensitivity (e.g. the ability to detect clinical levels of PTSD symptomatology) among civilian (Blevins, Weathers, Davis, Witte, & Domino, 2015), veteran (Bovin et al., 2016), and military populations (Wortmann et al., 2016).

*The Multiscale Dissociation Inventory* (MDI; Briere, 2002)

The MDI is a 30-item self-report inventory used to measure the frequency at which individuals experience six domains of dissociative symptoms over the past month with adequate-high internal consistency: disengagement (MDI disengagement; α = 0.83), depersonalization (MDI depersonalization; α = 0.90), derealization (MDI derealization; α = 0.91), emotional constriction (MDI emotional constriction; α = 0.94), memory disturbance (MDI memory disturbance; α = 74), and identity dissociation (MDI identity dissociation; α = 75). The MDI Total score has an internal consistency of α = 0.96.

*The Difficulties in Emotion Regulation Scale* (DERS; Gratz & Roemer, 2004)

The DERS assesses six dimensions of emotion regulation difficulties including, lack of awareness of emotional responses, lack of clarity of emotional responses, nonacceptance of emotional responses, limited emotion regulation strategies, difficulties controlling impulses when experiencing negative emotions, and difficulty completing goal-directed behaviours when experiencing negative emotions (Gratz & Roemer, 2004). The DERS has been found to have good internal consistency (α = 0.80–0.89 for subscales), convergent validity with other measures of emotion regulation, and predictive validity for behavioural outcomes including self-harm (Gratz & Roemer, 2004).

*The Toronto Alexithymia Scale* (TAS; Bagby, Taylor, & Parker, 1994)

The TAS is a 20-item self-report measure assessing the construct of alexithymia, or difficulties recognizing and naming emotions. The TAS measures three dimensions of alexithymia: difficulty identifying feelings, difficulty describing feelings, and externally-oriented thinking (i.e. the preference to focus on external details rather than internal thought content related to emotions). The TAS has demonstrated good test-retest reliability and internal consistency (α = 0.81) (Bagby et al., 1994).

*The Depression Anxiety and Stress Scale – 21-item version* (DASS-21; Lovibond & Lovibond, 1995)

The 21-item version of the DASS was used to measure symptoms of depression (DASS depression) (low mood, motivation and self-esteem), anxiety (DASS anxiety) (physiological arousal, panic, and fear), and stress (DASS stress) (tension and irritability). The DASS-21 has demonstrated good internal consistency (α = 0.81–0.91 for subscales) and convergent validity (Lovibond & Lovibond, 1995).

The *Adverse Childhood Experiences Questionnaire (ACE-Q)* (Felitti et al., 1998; Merrick et al., 2017) was used to assess the presence or absence of 10 commonly experienced ACEs. Specifically, using dichotomous variables (0 = No, 1 = Yes), participants indicated whether they had been exposed to emotional, physical, or sexual abuse, emotional or physical neglect, domestic violence (physical abuse of mother), parental divorce or separation, substance abuse in the household, a mentally ill family member, or incarceration of a family member. A cumulative index combining individual ACE-Q items results in a total exposure score ranging from 0–10. ACE-Q scores are related to adverse mental health outcomes including drug use, alcohol use, depressed affect and attempted suicide, with increasing ACE scores being related to increased odds of experiencing adverse outcomes (Merrick et al., 2017).

### 2.3. Procedures and statistical analyses

All analyses were completed using SPSS version 24.0. Analysis of the distribution of variables assessed in the current study revealed non-normality of several variables (Shapiro-Wilk >.05). Parametric analyses were reported for clarity and ease of interpretation. However, non-parametric tests (not reported) revealed consistent results across analyses.

Correlational analyses were conducted to examine the relation between PTSD symptomatology, dissociation, emotion regulation difficulties, alexithymia, depression, anxiety, and stress with functional impairment in the full sample (Pearson’s *r*; two-tailed; alpha = 0.05). Correlational analysis was also conducted to examine the relation between exposure to ACEs and functional impairment for those respondents with complete ACE-Q data.

Comparisons of means on clinical measures and functional impairment were made between the military/veteran (*n* = 32) and first responder (*n* = 27) groups. Group differences were analyzed using a univariate analysis of variance (ANOVA), which treated military/veteran and first responder status as fixed variables and the clinical measures as dependent variables.

Comparison of mean number of ACEs reported was made between the military/veteran (*n* = 30) and first responder (*n* = 26) groups for those respondents with complete ACE-Q data. Pearson’s chi-square tests were used to determine whether there was an association between military or first responder status and exposure to individual ACEs.

In order to test our hypothesis that dissociative symptoms (MDI Total and derealization score) mediate the relation between PTSD symptomatology (PCL-5 total score) and functional impairment, we followed the tests proposed by (Hayes, 2013) as well as the bootstrapping procedure (5000 samples) recommended by Preacher and Hayes (Preacher & Hayes, 2004). Mediation analyses were carried out using the using the PROCESS macro, version 2.16, for SPSS (Hayes, 2013). In order to account for the possibility that exposure to ACEs may be associated with functional impairment and thus account for the relation between PTSD symptomatology and functional impairment, secondary analyses were completed, repeating the above mediation analyses with ACE-Q Total score included as a covariate in the models. Critically, as Lees and Neufeld (1994) describe, while statistical control (such as adding covariates) can lead to issues such as data residualization and deconstruction leading to compromised meaning of the covariate-adjusted value, this approach is often the only option in clinical research. However, if the inferences drawn from our initial data analysis (e.g. the mediating role of dissociative symptoms) remain stable in our secondary analyses (including the covariate), we can be confident in our conclusions.

## 3. Results

### 3.1. Relation between functional disability (WHODAS Total) and variables of interest

Table 2 reports the correlations between functional disability and clinical variables as well as cumulative ACE exposure. Correlations are reported as statistically significant after controlling for multiple comparisons. Among the variables of interest, symptoms of dissociation (MDI Total) were most strongly correlated with functional impairment as measured by the WHODAS Total (*r* = 0.59, *p *< .002). In addition, in order of increasing strength of association, DERS Total (*r* = 0.50, *p *< .002), DASS depression (*r* = 0.47, *p *< .002), TAS Total (*r* = 0.43, *p *< .002), and DASS anxiety (*r* = 0.40, *p *< .002), were significantly related to functional impairment (WHODAS Total) (see Table 2). PTSD symptoms and cumulative ACE exposure were not significantly correlated with WHODAS Total after correcting for multiple comparisons.

**Table 2.** Correlations (Pearson’s *r*) between functional impairment and PTSD and associated symptom dimensions (*N =* 62 for all variables except for ACE-Q, *N =* 59).

	WHODAS Total
ACE-Q	0.27
PCL Total	0.30
PCL Intrusions	0.32
PCL Avoidance	0.16
PCL Cognition and Mood	0.23
PCL Arousal and Reactivity	0.30
MDI Total	0.59*
MDI Disengagement	0.57*
MDI Depersonalization	0.47*
MDI Derealization	0.59*
MDI Emotional Constriction	0.52*
MDI Memory Dissociation	0.50*
MDI Identity Dissociation	0.31
DASS-Depression	0.47*
DASS-Anxiety	0.40*
DASS-Stress	0.23
DERS Total	0.50*
TAS Total	0.43*

*Significant after controlling for multiple comparisons, *p *< .002;

ACE-Q, Adverse Childhood Experiences Questionnaire; PCL, PTSD Checklist For DSM-5; MDI, Multiscale Dissociation Inventory; DASS, Depression Anxiety Stress Scale; DERS, Difficulties in Emotion Regulation Scale; TAS, Toronto Alexithymia Scale.

Among MDI subscales, MDI derealization symptoms emerged as the most significant correlate with functional impairment (*r* = 0.59, *p *< .002). In addition, MDI disengagement (*r* = 0.57, *p *< .002), MDI emotional constriction (*r* = 0.52, *p *< .002), MDI memory dissociation (*r *= 0.50, *p *< .002), and MDI depersonalization (*r* = 0.47, *p *< .002) were significantly related to functional impairment.

### 3.2. Group comparisons for results on clinical measures and exposure to ACEs

Table 3 reports the means, standard deviations, and group comparisons for clinical measures as well as percentage of the sample endorsing each ACE-Q and Pearson’s chi-square test of association. Compared to first responders, the military/veteran group endorsed significantly higher scores on measures of functional impairment (*F* (1,58) = 15.15, *p* = .000), derealization symptoms (*F* (1,58) = 4.17, *p* = .046), alexithymia (*F* (1,58) = 4.29, *p* = .043), depression (*F* (1,58) = 4.09, *p* = .048), and anxiety (*F* (1,58) = 4.16, *p* = .046). No other significant differences emerged.

**Table 3.** Clinical characteristics of study sample and comparison of clinical measures and exposure to ACEs between military members/veterans and first responders.

	Combined sample, *n* = 62	Military and veteran, *n* = 32	First Responder, *n* = 27	*F* (1,58)
*Clinical Characteristics*	Mean (*SD*)			
Functional Disability (WHODAS 2.0)	53.9 (18.2)	62.3 (14.2)	45.8 (18.3)	15.15**
PTSD Symptoms (PCL-5)				
Total	59.5 (10.3)	59.8 (10.9)	59.8 (9.8)	0.00
Intrusions	14.5 (4.2)	14.8 (4.6)	14.7 (3.7)	0.00
Avoidance	6.1 (1.8)	6.1 (1.6)	6.3 (2.0)	0.05
Cognition and Mood	21.5 (3.8)	21.4 (4.0)	21.2 (3.5)	0.02
Arousal and Reactivity	17.1 (3.4)	17.5 (3.5)	21.2 (3.5)	0.37
Dissociative Symptoms (MDI)				
Total	69.5 (25.4)	75.4 (27.9)	65.1 (21.3)	2.48
Disengagement	16.3 (4.9)	17.0 (4.7)	16.1 (5.2)	0.44
Depersonalization	8.9 (4.9)	10.0 (5.5)	8.1 (4.1)	2.33
Derealization	11.5 (5.2)	12.9 (5.4)	10.2 (4.8)	4.17*
Emotional Constriction	14.0 (6.0)	15.3 (6.1)	13.3 (5.5)	1.64
Memory Dissociation	12.2 (5.6)	13.1 (6.2)	11.6 (4.8)	1.19
Identity Dissociation	6.5 (3.1)	7.0 (3.9)	5.8 (1.7)	2.33
Emotion Dysregulation (DERS)	119.5 (24.3)	123.8 (27.9)	116.9 (22.1)	1.18
Alexithymia (TAS)	63.4 (12.3)	66.3 (13.6)	59.7 (9.9)	4.29*
DASS-Depression	27 (10.4)	29.8 (10.6)	24.4 (9.6)	4.09*
DASS-Anxiety	23.4 (10.1)	25.8 (9.4)	20.6 (10.2)	4.16*
DASS-Stress	27.5 (8.5)	27.8 (7.8)	27.5 (9.5)	0.02
ACE-Q Total	3.36 (2.73)	3.67 (2.70)	3 (2.79)	0.83
ACE-Q (*n = *59; *n* = 30 Military/Veteran; *n* = 26 First Responder)	% of Sample Endorsing			Χ^2^(2)
Emotional Abuse	57.6	60	53.8	0.22
Physical Abuse	50.8	53.3	50	0.06
Sexual Abuse	23.7	33.3	15.4	2.39
Emotional Neglect	38.9	36.7	42.3	0.19
Physical Neglect	18.6	23.3	15.4	0.56
Parental Separation/Divorce	40.7	43.3	34.6	0.44
Domestic Violence	23.7	33.3	11.5	3.71
Substance Abuse	39.0	40	38.5	0.01
Mental Illness	33.9	36.7	30.8	0.22
Incarceration of household member	6.8	6.7	7.7	0.02

*p < .05, ** *p < *.01.

PCL, PTSD Checklist For DSM-5; MDI, Multiscale Dissociation Inventory; DASS, Depression Anxiety Stress Scale; DERS, Difficulties in Emotion Regulation Scale; TAS, Toronto Alexithymia Scale; ACE-Q, Adverse Childhood Experiences Questionnaire.

*F* refers to *F* statistic for univariate analysis of variance; Χ^2^ refers to the chi-square statistic for Pearson’s chi-square test.

No significant associations emerged between military/veteran or first responder status and exposure to individual ACEs. There was a trend-level association between military/veteran or first responder status and exposure to domestic violence (i.e. witnessing abuse of mother), Χ^2^(2) = 3.71, *p* = .054, with 33.3% military/veterans compared with 11.5% of first responders endorsing exposure to domestic violence in childhood.

### 3.3. Mediation of association between PTSD symptom severity and functional disability by dissociative symptoms

Given the strong correlation between MDI Total and WHODAS Total, as well as the theoretical rationale outlined above, we examined whether dissociative symptoms (MDI Total) mediated the relation between PTSD severity (PCL Total) and functional impairment (WHODAS Total). In addition, we investigated whether dissociative symptoms of derealization, in particular, were driving the relation between PTSD severity and functional impairment as, among subscales of the MDI, the derealization subscale emerged as the strongest correlate of functional impairment, and given that it represents an important symptom of PTSD+DS. Tests for mediation were carried out according to Hayes (2013). The proportion of variance accounted for by the mediation model is also provided. Although some of the data in the current study were not normally distributed (Shaprio-Wilk < .05), bootstrapping techniques as followed in the present analyses are robust to violations of the assumptions of normality and homoscedasticity (Erceg-Hurn & Mirosevich, 2008).

#### 3.3.1. Mediation the of association between PTSD symptom severity and functional disability by MDI Total

**Figure 1.**
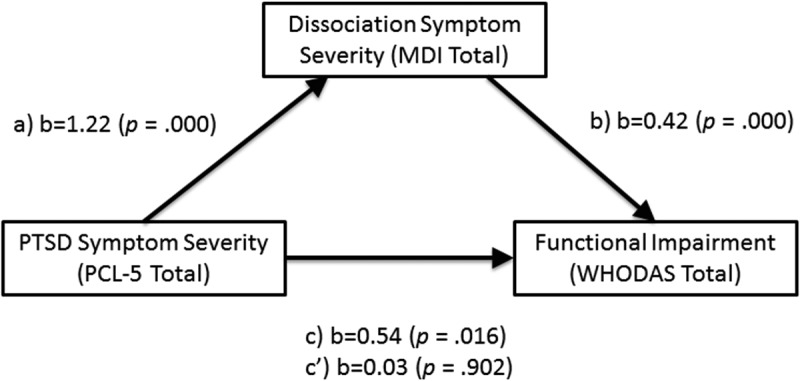
Depiction of the mediation model where dissociative symptoms (MDI Total) mediate the relation between PTSD severity and functional impairment. The effect of PCL Total on change in WHODAS Total when MDI Total is introduced as a mediator (c’) is nonsignificant. b = unstandardized coefficients; a = effect of PCL Total on change in MDI Total; b) effect of MDI Total on change in WHODAS Total; c = the total effect; c’ = the direct effect MDI, multiscale dissociation inventory; PCL-5, PTSD checklist for DSM-5; WHODAS, World Health Organization Disability Assessment Schedule 2.0.

Table 4 reports the correlations between the variables in the mediation model. Table 5 and Figure 1 summarize the results of the mediation analysis. Results indicated that PTSD symptom severity was a significant predictor of dissociative symptoms, b = 1.22, *SE* = 0.28, *p* = .000, and that dissociative symptoms significantly predicted functional impairment, b = 0.42, *SE = *0.09, *p* = 000. PTSD symptoms were no longer a significant predictor of functional impairment after controlling for dissociative symptoms, b = 0.03 *SE *= 0.21, *p* = .902. Approximately 35% of the variance in functional impairment was accounted for by the predictors (R^2^ = 0.35). The indirect effect was estimated using a bootstrapping approach with 5000 samples. These results indicated the indirect coefficient was significant, b = 0.51, *SE *= 0.13, *p* = 001. For every 1-point increase in PTSD symptom severity, there was an approximately 0.51-point increase in functional impairment, as mediated by dissociative symptoms.

**Table 4.** Correlation matrix for dissociation symptom severity (MDI Total), PTSD symptom severity (PCL Total), and functional disability (WHODAS Total), *N* = 62 (Pearson’s *r* reported).

Variable	2	3
1. MDI Total	0.50***	0.59***
2. PCL Total		0.30*
3. WHODAS Total		

*p < .05; **p < .01; ***p < .001.

PCL, PTSD Checklist For DSM-5; MDI, Multiscale Dissociation Inventory; WHODAS. World Health Organization Disability Assessment Schedule 2.0.

**Table 5.** Mediation effects of dissociation symptom severity (MDI Total) on the relationship between PTSD symptom severity (PCL Total) and functional disability (WHODAS Total), *N *= 62.

Regression Paths	*b*	SE	*p*
Mediation *a* path (PCL Total on MDI Total)	1.22	0.28	< .001
Mediation *b* path (MDI Total on WHODAS Total)	0.42	0.09	< .001
Total effect, *c* path (PCL Total on WHODAS Total, No Mediator)	0.54	0.22	< .05
Direct effect *c’* (PCL Total on WHODAS Total including MDI Total as mediator)	0.03	0.21	0.902
Indirect effect bootstrapped (*c – c’*) with bootstrapped 95% CI	0.51 [0.27–0.79]		

*b* = unstandardized coefficient; SE = standard error; CI = confidence interval. Fit for mediation model *R^2^ = *0.35, *F* (2, 59) = 15.83, *p *< .001.

PCL, PTSD Checklist For DSM-5; MDI, Multiscale Dissociation Inventory; WHODAS, World Health Organization Disability Assessment Schedule 2.0.

Inclusion of ACE-Q total as a covariate in a secondary mediation analysis completed on respondents with full ACE-Q data available (*n* = 59) revealed consistent results. Specifically, PTSD symptom severity was a significant predictor of dissociative symptoms after controlling for cumulative ACEs, b = 1.26, *SE* = 0.33, *p* = .000, and dissociative symptoms significantly predicted functional impairment after controlling for cumulative ACEs, b = 0.36, SE = 0.10, *p* = .000. PTSD symptoms were no longer a significant predictor of functional impairment after controlling for dissociative symptoms and cumulative ACEs, b = 0.11 (*SE *= 0.22), *p* = .646. Approximately 38% of the variance in functional impairment was accounted for by the predictors (R^2^ = 0.38). The indirect effect was estimated using a bootstrapping approach with 5000 samples. These results indicated the indirect coefficient was significant, b = 0.46, *SE *= 0.18, *p* = 008.

#### 3.3.2. Mediation of association between PTSD symptom severity and functional disability by MDI derealization symptoms

**Figure 2.**
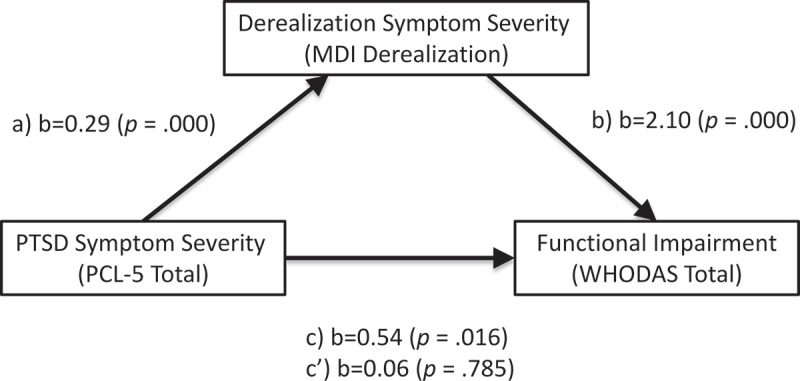
Depiction of the mediation model where derealization symptoms (MDI derealization) mediate the relation between PTSD severity (PCL Total) and functional impairment (WHODAS Total). The effect of PCL Total on change in WHODAS Total when MDI derealization is introduced as a mediator (c’) is nonsignificant. b = unstandardized coefficients; a = effect of PCL Total on change in MDI derealization; b) effect of MDI derealization on change in WHODAS Total; c = the total effect; c’ = the direct effect MDI, Multiscale dissociation inventory; PCL-5, PTSD Checklist for DSM-5; WHODAS, World Health Organization Disability Assessment Schedule 2.0.

Table 6 depicts the correlations between the variables in the mediation model. Table 7 and Figure 2 summarize the results of the mediation analysis. Results indicated that PTSD symptom severity was a significant predictor of derealizsation symptoms, b = 0.29, *SE* = 0.05, *p* = .000, and that derealizsation symptoms significantly predicted functional impairment, b = 2.10, *SE = *0.44, *p* = 000. PTSD symptoms were no longer a significant predictor of functional impairment after controlling for derealizsation symptoms, b = −0.06, *SE *= 0.22, *p* = 0.785. Approximately 34% of the variance in functional impairment was accounted for by the predictors (R^2^ = 0.34). The indirect effect was estimated using a bootstrapping approach with 5000 samples. These results indicated the indirect coefficient was significant, b = 0.60, *SE *= 0.14, *p* = 001. For every 1-point increase in PTSD symptom severity, there was an approximately 0.60-point increase in functional impairment, as mediated by derealization symptoms.

**Table 6.** Correlation matrix for derealization symptoms (MDI derealization), PTSD symptom severity (PCL Total), and functional disability (WHODAS Total), *N* = 62 (Spearman’s rho reported).

Variable	2	3
1. MDI Derealization	0.56***	0.59***
2. PCL Total		0.30*
3. WHODAS Total		

*p < .05; **p < .01; ***p < .001.

PCL, PTSD Checklist For DSM-5; MDI, Multiscale Dissociation Inventory; WHODAS, World Health Organization Disability Assessment Schedule 2.0.

**Table 7.** Mediation effects of derealization symptoms (MDI derealization) on the relationship between PTSD symptom severity (PCL Total) and functional disability (WHODAS Total), *N *= 62.

Regression Paths	*b*	SE	*p*
Mediation *a* path (PCL Total on MDI Derealization)	0.29	0.05	< .001
Mediation *b* path (MDI Derealization on WHODAS Total)	2.10	0.44	< .001
Total effect, *c* path (PCL Total on WHODAS Total, No Mediator)	0.54	0.22	< .05
Direct effect *c’* (PCL Total on WHODAS Total including MDI Derealization as mediator)	−0.06	0.22	0.785
Indirect effect bootstrapped (*c – c’*) with bootstrapped 95% CI^b^	0.60 [0.33–0.89]		

*b* = unstandardized coefficient; SE = standard error; CI = confidence interval. Fit for mediation model *R^2^ = *0.34, *F* (2, 59) = 15.40, *p *< .001.

PCL, PTSD Checklist For DSM-5; MDI, Multiscale Dissociation Inventory; WHODAS, World Health Organization Disability Assessment Schedule 2.0.

Inclusion of ACE-Q total as a covariate in a secondary mediation analysis completed on respondents with full ACE-Q data available (*n* = 59) revealed consistent results. Specifically, PTSD symptom severity was a significant predictor of dissociative symptoms after controlling for cumulative ACEs, b = 1.26, *SE* = 0.33, *p* = .000, and derealization symptoms significantly predicted functional impairment after controlling for cumulative ACEs, b = 1.85, SE = 0.46, *p* = .000. PTSD symptoms were no longer a significant predictor of functional impairment after controlling for dissociative symptoms and cumulative ACEs, b = 0.04 (*SE *= 0.26), *p* = .893. Approximately 37% of the variance in functional impairment was accounted for by the predictors (R^2^ = 0.37). The indirect effect was estimated using a bootstrapping approach with 5000 samples. These results indicated the indirect coefficient was significant, b = 0.53, *SE *= 0.14, *p* = 003.

## 4. Discussion

This study is the first to explicitly examine the mediating role of dissociative symptoms in the relation between PTSD symptom severity and functional impairment. Here, we found that dissociative symptoms experienced over the past month, as measured by the MDI (Briere, 2002), significantly mediated the relation between PTSD symptom severity (as measured by the PCL-5) and functional impairment (as measured by the WHODAS 2.0) among a sample of military members, veterans, and first responders. Given that the highest correlation among MDI subscales and functional disability emerged for the derealization subscale and the role of derealization in PTSD+DS, we investigated whether derealization symptoms mediated the relation between PTSD severity and functional disability and found a significant mediation effect. The emergence of derealization symptoms as a significant mediator is interesting given previous findings that derealization (but not depersonalization) was associated with increased disease severity among individuals with trauma-related disorders (e.g. dissociative disorders and borderline personality disorder; Sar, Alioğlu, & Akyuz, 2017), suggesting that derealization and depersonalization symptoms represent distinct constructs with different properties. These results support previous literature highlighting increased disease severity and functional impairment among individuals with PTSD+DS (Evren et al., 2011; Stein et al., 2013), including military members and veterans (Tsai et al., 2015; Waelde et al., 2005; Wolf et al., 2012, 2012).

In the current sample, PTSD symptoms were not significantly correlated with functional impairment after controlling for multiple comparisons, despite being correlated with other symptoms found to be significantly correlated with functional impairment (not reported), including dissociative symptoms, depression, emotion regulation difficulties, anxiety, and alexithymia. Thus, it may be important for treatment to target these additional symptom domains in order to achieve functional recovery. These findings contrast with previous work identifying avoidance/numbing (Rona et al., 2009; Shea et al., 2010) and hyperarousal symptoms (Heir et al., 2010; Maguen et al., 2009) as being significantly related to functional impairment among individuals with PTSD. To our knowledge, this is the first study to investigate DSM-5 PTSD symptom dimensions in relation to functional impairment, which may account for these discrepant findings, given the differences in PTSD symptom domains between DSM-5 and DSM-IV-TR.

The current study is in keeping with findings of increased cognitive dysfunction among individuals with PTSD and dissociative symptoms (McKinnon et al., 2016), including veterans (Morgan et al., 2006; Roca et al., 2006). In particular, we hypothesize that symptoms of dissociation may lead to increased functional impairment at least partially via their relation with increased cognitive dysfunction. Here, given limited cognitive processing resources, it is probable that dissociative symptoms may reduce the amount of resource available to key cognitive domains, including attention and executive functioning, resulting in functional limitations (McKinnon et al., 2016). Indeed, among veterans with PTSD, reduced verbal memory performance has been associated with reduced social and occupational functioning (Geuze et al., 2009; but see Twamley et al., 2009). Future work will be necessary to confirm this hypothesis.

Notably, previous work has highlighted that among a subgroup of men seeking treatment for alcohol abuse who had PTSD, the presence of dissociative symptoms was related to reduced quality of life. In addition, dissociative symptoms mediated the relation between childhood trauma and PTSD severity (Evren et al., 2011). Given these findings, and findings of high levels of childhood abuse among military members and veterans when compared with the general population (Afifi et al., 2016; Koola et al., 2013; Seifert, Polusny, & Murdoch, 2011), it is possible that dissociative symptoms may stem, in part, from childhood abuse rather than trauma experienced as part of a military or first responder service, thus leading to functional impairment. However, the results of the present study indicated that dissociative symptoms mediated the relation between PTSD symptoms and functional impairment after controlling for exposure to ACEs, suggesting that while ACEs may be associated with functional impairment, they do not account for the mediating role of dissociative symptoms.

Our findings support the critical importance of identifying dissociative symptoms when assessing and diagnosing PTSD. In particular, the present results suggest that treatment targeting dissociative symptoms, particularly derealization symptoms, among military members and first responders may be imperative in allowing both symptomatic and functional recovery from PTSD. Given that recent studies have identified that 8–32% of veterans and military members with PTSD can be classified as a dissociative subtype (PTSD+DS) (Armour et al., 2014; Tsai et al., 2015; Waelde et al., 2005; Wolf et al., 2012, 2012), these findings hold particular importance for these populations. Critically, military members and veterans often struggle to shift from military or service life to other roles, a transition that is potentially related to the high levels of functional impairment seen in the current sample. Moreover, some findings indicate that dissociative symptoms may impact negatively on treatment outcome with first-line treatments for PTSD such as, eye movement desensitization and reprocessing (EMDR) (Bae, Kim, & Park, 2016), cognitive processing therapy (CPT) (Resick, Suvak, Johnides, Mitchell, & Iverson, 2012), and early intervention using imaginal and in-vivo exposure techniques (Price, Kearns, Houry, & Rothbaum, 2014; Wolf, Lunney, & Schnurr, 2016). Although some authors questions the clinical significance of these findings (Resick et al., 2012; Wolf et al., 2016) and others studies have not found reduced treatment efficacy among individuals with PTSD demonstrating significant dissociative symptoms (Cloitre, Petkova, Wang, & Lu, 2012; Hagenaars, Van Minnen, & Hoogduin, 2010; Speckens, Ehlers, Hackmann, & Clark, 2006; Wolf et al., 2016), individuals with PTSD+DS may be vulnerable to ongoing symptoms and disrupted functioning following treatment. Accordingly, alternative or adjunctive treatment strategies targeting symptoms of dissociation may be necessary to achieve both full symptomatic and functional recovery (Lanius, 2015). This is particularly relevant given work indicating that functional impairment may persist beyond symptom recovery in individuals with PTSD (Westphal et al., 2011). For example, mindfulness-based treatments have emerged recently as a promising alternative approach to treatment of PTSD symptomatology (Boyd, Lanius, & McKinnon, 2017; Hopwood & Schutte, 2017) and have been suggested to be an effective approach in the treatment of dissociative symptoms, whereby mindfulness based approaches foster increased connection and awareness to somatic experiences and awareness of the internal and external cues (Boyd et al., 2017; Zerubavel & Messman-Moore, 2015).

The current sample included first responders, military members, and veterans with PTSD. Although PTSD+DS has been studied among military members and veterans, it remains understudied among first responders. Indeed, just one study to date has identified significant dissociative symptoms among first responders (Carlier et al., 1996). In the present study, military members and veterans demonstrated dissociative symptoms at or above clinically significant levels (Briere, 2002) across symptom clusters of disengagement, depersonalization, derealization, emotional constriction, and memory dissociation, on average. In contrast, the first responder group demonstrated dissociative symptomatology at or above clinically significant levels (Briere, 2002) only on measures of disengagement, emotional constriction, and memory dissociation. Moreover, when comparisons were made between groups, the military and veteran group demonstrated significantly higher scores on the derealization subscale of the MDI. With estimates of 8–22% of first responders meeting criteria for PTSD (Andrews, Joseph, Shevlin, & Troop, 2006; Bennett, Williams, Page, Hood, & Woollard, 2004; Clohessy & Ehlers, 1999; Jonsson, Segesten, & Mattsson, 2003; Pietrzak et al., 2014), it is important for future work to identify the extent to which first responders meet criteria for the dissociative subtype. The present findings indicate that while dissociative symptoms may be present among first responders, they may be at a lower level than those seen in military members or veterans. Importantly, the military and veteran group also reported significantly higher levels of functional impairment than the first responder group, suggesting that higher levels of derealization symptoms in the military/veteran group may lead to higher levels of functional impairment, when compared with the first responder group. Given the small sample size in the present study (*n* = 32 military members and veterans; *n* = 27 first responders), these findings will need to be replicated in future studies with larger samples. This is particularly important given the present findings of a strong relation between dissociative symptoms and functional disability in a sample including first responders.

Additional comparisons between the military member and veteran group and first responder group revealed that both groups exhibited similar levels of PTSD severity. In contrast, the military member and veteran group endorsed significantly greater depressive symptoms, anxiety symptoms, and alexithymia. These findings support previous literature indicating high comorbidity of depression and PTSD in veterans, which is associated with reduced quality of life and greater symptom severity (Ginzburg, Ein-Dor, & Solomon, 2010; Ikin, Creamer, Sim, & McKenzie, 2010), complementing findings of greater functional impairment in the military/veteran group than the first responder group in the current study. Moreover, the finding of greater alexithymia among the military member and veteran group converges with previous findings demonstrating higher levels of alexithymia in combat-related PTSD compared to other trauma types (Frewen, Dozois, Neufeld, & Lanius, 2008). Levels of exposure to ACEs were also compared between the military/veteran and first responder groups. Interestingly, in the current sample military members and veterans reported an average of 3.7 ACEs and first responders reported an average of 3.0, a difference that was not statistically significant. In addition, no significant associations were found between military/veteran or first responder status and exposure to individual ACEs, although higher rates were reported among military members and veterans for experiencing sexual abuse (33.4% vs. 15.4%) and domestic violence (33.3% vs. 11.5%). Future work with larger sample sizes and utilizing community samples will be necessary to determine if there is a statistically significant difference in exposure rates.

Overall, the current study provides preliminary, but provocative, evidence that dissociative symptoms mediate the relation between PTSD symptomatology and functional impairment among veterans, military members, and first responders with PTSD. It will be critical to investigate whether this mediating relation holds true for other populations with PTSD (e.g. childhood abuse survivors). Furthermore, the current study assessed PTSD symptoms over the past month, but did not assess chronic or persistent PTSD symptomatology. Previous work indicates that lower levels of psychosocial functioning is associated with maintenance of chronic PTSD (Zlotnick et al., 2004). Given the inpatient status of the current sample, it is likely that they have experienced a persistent and severe course of illness, consistent with a chronic presentation. Here, future work is required to evaluate the effect of course of illness (e.g. duration, severity) on the relation between chronicity of PTSD, dissociative symptoms, and functional impairment, and to determine if these findings will generalize to other groups (e.g. community samples, those seeking outpatient treatment). In addition, future work clarifying whether individuals with PTSD+DS exhibit greater functional impairment than those with PTSD. Further, it will be important to determine whether differing mediating relations emerge among those with PTSD who meet criteria for the dissociative subtype and those who do not. In particular, prospective studies should identify which symptom domains are most important in predicting functional impairment among those with PTSD+DS and PTSD.

There are several limitations to the present study that may be addressed in future research. The sample size of the current study is low and only self-report, retrospective questionnaires were used, along with best estimates of PTSD diagnosis based on self-report. Future work should utilize more rigorous psychodiagnostic measures to confirm a diagnosis of PTSD and to assess for the presence and severity of related symptoms, including dissociation. Furthermore, our study was limited to first responders, veterans, and military members, was largely composed of males, and may not generalize to other trauma-exposed populations or females with PTSD. In addition it was not possible to determine the traumatic experience from which participants in the current sample were experiencing the most distress (e.g. military or combat trauma or childhood abuse), thus although inclusion of ACEs as a covariate in our analyses did not affect the results, it is possible that subjects in this study were experiencing distress related to other traumatic experiences in addition to military/combat trauma or their work as first responders. Similarly, due to the potential overlap of symptoms due to different traumatic experiences (e.g. childhood abuse and combat trauma) it is possible that the mediating role of dissociation between PTSD and functional impairment is not accounted for solely by participant’s combat or first responder experiences. We were also unable to account for a history physical injury, traumatic brain injury, or other physical illnesses that may be associated with functional impairment and dissociative symptoms. This is particularly important given recent findings that physical injury may be associated with increased dissociative symptoms among combat-exposed individuals with PTSD (Özdemir, Celik, & Oznur, 2015). Future studies should exclude individuals with these medical histories in order to ascertain a clearer picture of the relation between dissociation, PTSD symptoms, and functional impairment. The use of a cross-sectional design also limits the conclusions that can be drawn. Specifically, since all measures were collected at the same time, conclusions regarding causality are limited and it is difficult to determine if dissociative symptoms predict or mediate the relation between PTSD symptoms and functional impairment over time. The use of longitudinal designs in future research will be necessary to draw such conclusions. Moreover, given that PTSD symptoms and dissociative symptoms are highly correlated, and some models of dissociative symptoms in PTSD suggest that they both represent aspects of the same response to traumatic events (Dalenberg & Carlson, 2012), it is possible thatm removing the variance associated with dissociative symptoms in the mediation analysis will also remove variance associated with PTSD, thus erroneously reducing the significance of PTSD symptoms as predictors of functional outcomes.

Nonetheless, enhanced knowledge of the relation between PTSD and functional impairment and the mediating role of dissociative symptoms, particularly derealization, is critical in assisting clinicians in understanding which symptoms must be targeted in order to achieve both functional and symptomatic recovery among individuals with PTSD.
